# The yellow brick road to nuclear membrane mechanotransduction

**DOI:** 10.1063/5.0080371

**Published:** 2022-03-25

**Authors:** Zhouyang Shen, Miklós Lengyel, Philipp Niethammer

**Affiliations:** 1 Cell Biology Program, Memorial Sloan Kettering Cancer Center, New York, New York 10065, USA; 2 Gerstner Sloan Kettering Graduate School of Biomedical Sciences, New York, New York 10065, USA

## Abstract

The nuclear membrane may function as a mechanosensory surface alongside the plasma membrane. In this Review, we discuss how this idea emerged, where it currently stands, and point out possible implications, without any claim of comprehensiveness.

## INTRODUCTION

When the body of a eukaryotic cell is critically swollen, stretched, or compressed by cell-external or -internal forces, odds are that the nucleus, its largest and most central organelle, deforms accordingly. Coupling nuclear deformation to the activation of chemical signaling circuits allows a cell to detect mechanical forces on its center and distinguish them from forces on its periphery such as growth cones or other types of motile protrusions. Thereby, cells can adapt their acute behaviors and long-term fate to their physical environment and rapidly respond to changes within it.

The nucleus is a composite structure made up of three mechanosensitive components:^1^ the chromatin that it houses, the nuclear lamina that stiffens it, and the nuclear membrane (NM) (inner and outer) that serves as a permeability barrier to allow controlled nuclear-cytoplasmic trafficking. Upon strong nuclear deformation, each of these components experience structural changes that can cause masking or unmasking of molecular interaction sites. Mechanically induced loss or gain of molecular interactions inhibits or activates biochemical reactions and gene transcription, respectively. Here, we offer our perspective on how the idea of NM-mechanotransduction emerged as a physiologically relevant principle, summarize what is known about its mechanisms, and speculate about its possible implications.

## THE WONDERFUL MECHANISM OF OS

Research into NM-mechanotransduction, initially unaware of itself, started about ten years ago with a curious observation. In zebrafish, which live in fresh water, cells close to epithelial wound sites will experience a hypotonic shock. Nicking zebrafish larvae with a tungsten needle at their tail fin usually provokes rapid migration of neutrophils to the injury site within minutes. However, when larvae are wounded in an isotonic solution that mimics the much higher osmolarity of their interstitial fluid, wound detection by neutrophils is strongly suppressed.^2^ This osmotic surveillance (OS) mechanism mediates rapid immune detection of wounds and microbial infection.^3^ Zebrafish with inactivated OS are less likely to survive infections outlining a potential rationale for the evolutionary selection of OS as an epithelial breach detection mechanism of freshwater fish. Interestingly, the mucosal linings of our upper digestive tract are flushed with more than a liter of fresh water, i.e., saliva, each day. When we bite our tongue or cheeks, the skin heals quickly and rarely develops serious infections despite the presence of potentially harmful bacteria in our mouth. Saliva is a critical component of oral immune defense: If its production is perturbed, such as in Sjögren's syndrome, increased microbial infections are common.^4^ For reasons that are poorly understood, our bodies invest a great deal of energy to desalt the initially isotonic saliva to cover the upper digestive tract linings with hypotonic solution. It seems worthwhile to test whether these areas of skin promote wound detection as it has been shown in zebrafish larvae.

The molecular mechanism of OS involves the osmotic activation of an important lipid hormone pathway, the eicosanoid cascade, which converts arachidonic acid (AA) by enzymatic oxidation into a diverse set of bioactive lipids that comprise prostaglandins, leukotrienes, and many others. One of those eicosanoids, 5-oxoETE, mediates rapid wound detection by zebrafish neutrophils downstream of OS.^2^ The rate-limiting step of eicosanoid synthesis is the release of AA from NM- or endoplasmic reticulum (ER) -phospholipids catalyzed by Ca^2+^-dependent cytosolic phospholipase A2 (cPLA_2_, PLA2G4A). In mammalian tissues, inactive cPLA_2_ is typically located in the cytoplasm and, albeit more rarely, in the nucleoplasm (e.g., in human urothelium^5^). Zebrafish cPla_2_ (Pla2g4aa) appears to be mostly nucleoplasmic.^2^ The mechanisms that regulate cPLA_2_'s nuclear localization are largely unknown, although hitchhiking on chromatin regulators, such as PLIP (“cPLA_2_ interacting protein” = splice variant of lysine acetyltransferase 5/KAT5), might play a role.^6^ Depending on whether cPLA_2_ is initially localized to the cyto- or nucleoplasm, it binds to the outer or inner NM (ONM/INM) upon activation. Membrane recruitment is mediated by cPLA_2_'s C2-domain, which requires Ca^2+^ to neutralize negative membrane repulsion.

## A NEW TYPE OF MEMBRANE TENSION SENSOR

There have been several hints toward a mechanosensitivity of the eicosanoid cascade: prostaglandin E2 (PGE_2_), which is synthesized by cyclooxygenases from AA, is thought to regulate load-dependent bone homeostasis.^7–15^ Several PLA_2_ isoforms, including cPLA_2_, have been implicated in osmotic cell volume regulation.^16^ *In vitro*, the membrane interactions and activities of various peripheral membrane enzymes, such as protein kinase C, protein lipase C, and snake venom PLA_2_, have long been known to depend on the lateral lipid pressure, which is decreased by stretch.^17–20^ Nevertheless, the physiological relevance of these observations has remained elusive for decades.

Hypotonic shock causes rapid nuclear swelling.^21–25^ The resulting nuclear deformation is essential for cPla_2_ activation and wound detection. Strong wound- or ionophore-induced cytoplasmic Ca^2+^ transients alone are not sufficient to activate the mechanism.^2,25^ Pharmacologic and genetic inhibition of cPla_2_ or 5-lipoxygenase (Alox5a), which helps to convert AA into chemotactic lipids, inhibited neutrophil recruitment. Conversely, AA applied to tail fins by bathing or micropipette patching reconstituted the inflammatory response, underlining the crucial role of the eicosanoid cascade for wound detection in zebrafish.^2,25,26^ Osmotically induced INM-adsorption of zebrafish cPla_2_ was observed around wounds in live zebrafish, as well as in permeabilized HeLa cells whose nuclei were swollen by colloid osmotic shock in the presence of Ca^2+^. Squeezing the nuclei of permeabilized cells with an agarose pad also induced cPla_2_-binding to the INM. As chemical signaling cascades are disrupted by cell permeabilization, the idea emerged that OS must present a simple physical process—perhaps the first described instance of physiological NM-mechanotransduction. The direct mechanosensitivity of cPla_2_ became clearer when its fluorescently tagged C2 domain was reconstituted together with Ca^2+^ ions and sugar-filled giant unilamellar vesicles (GUVs) that were osmotically swollen by immersion in a hypotonic sugar solution: Despite an abundance of free Ca^2+^, cPLA_2_-C2 barely bound to relaxed vesicles. Only when the vesicles were swollen did the domain start to adsorb, but it immediately dissociated from the GUV bilayers once the vesicles ruptured and relaxed.^25,27^ Hence, on the molecular level, OS is mediated by membrane tension dependent protein adsorption.

**FIG. 1.**
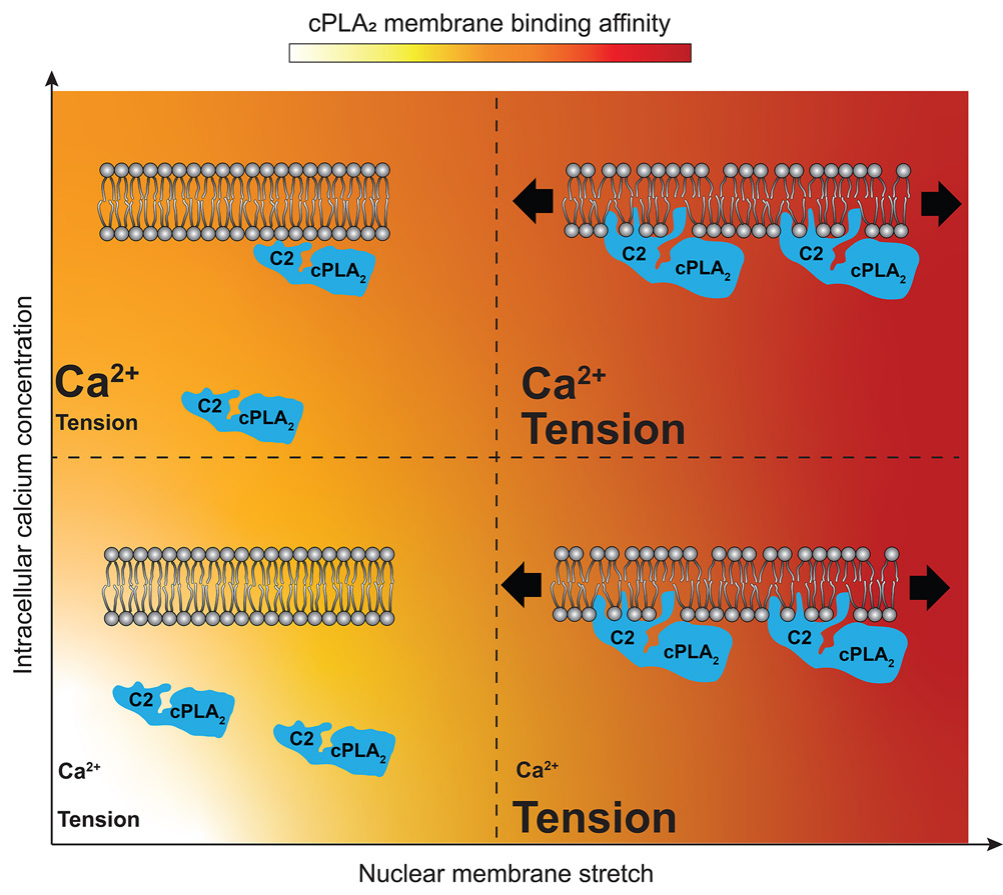
A synergy between mechanosensitive calcium- and membrane-binding mediates NM-tension-sensing by cPLA_2_. The tenser the NM, the less Ca^2+^ is required for efficient cPLA_2_ adsorption (red color gradient). In the extreme (right, lower corner), NM-stretch virtually replaces Ca^2+^ transients as primary cPLA_2_ activation signal through rendering the enzyme sensitive to resting Ca^2+^ concentrations. For further explanations, please see the main text.

New findings have shown that cPLA_2_ owes its exceptional tension sensitivity to conserved hydrophobic patches in its C2 domain.^27^ Penetrating the hydrophobic acyl core of the lipid bilayer allows protruding hydrophobic residues to synergize with Ca^2+^ to mediate order-of-magnitude increases in membrane binding affinity and calcium sensitivity of cPla_2_ on stretched vs unstretched GUVs or NMs, respectively (Fig. 1). Strong NM-stretch causes cPLA_2_ membrane adsorption even at resting Ca^2+^ concentrations (i.e., [Ca^2+^] <100 nM). In other words, in principle, tension transients can fully replace Ca^2+^ transients as the primary cPLA_2_ activation signal. However, as nuclear deformation is usually associated with cytoplasmic Ca^2+^ influx (see below), it remains unclear how relevant such a scenario is *in vivo*.

**FIG. 2.**
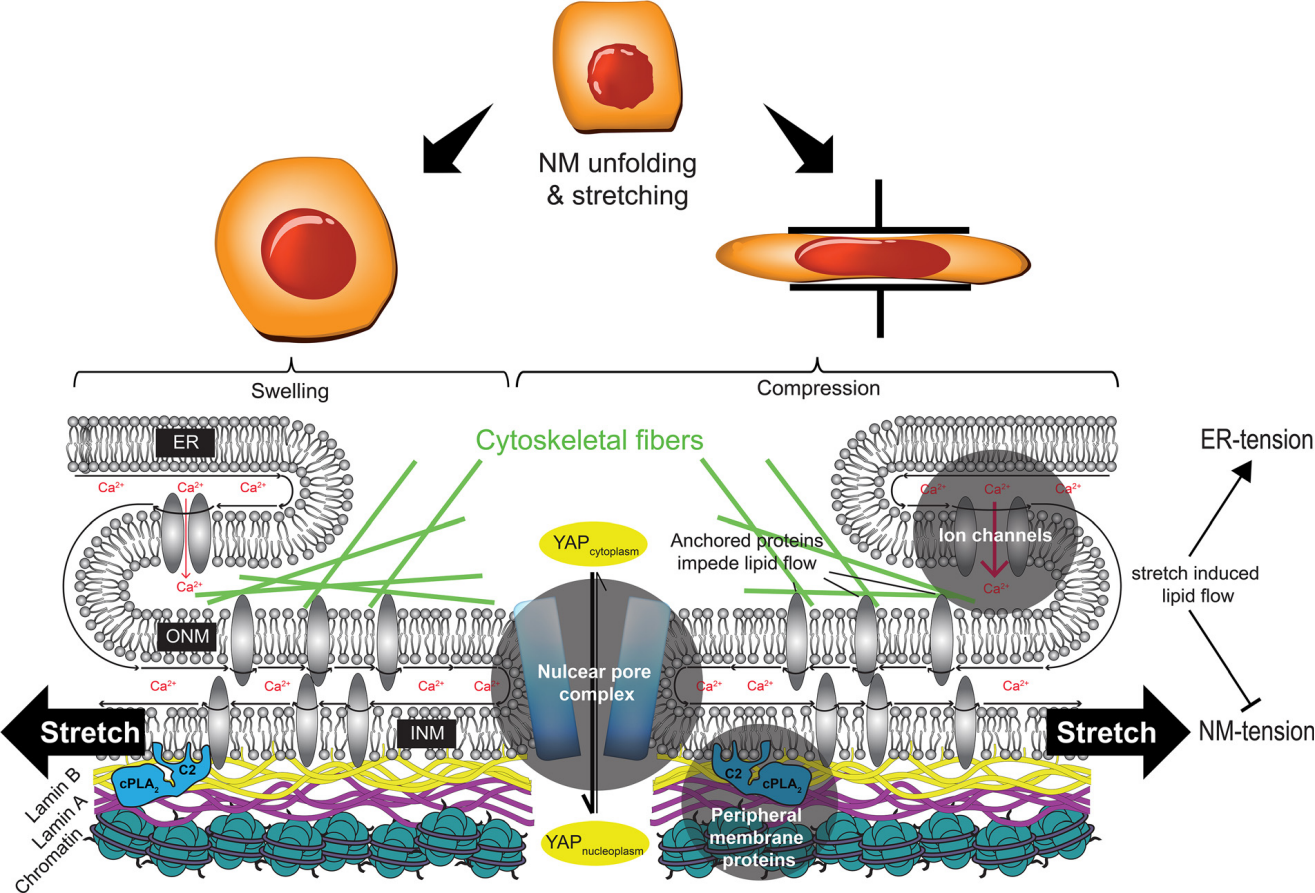
Hypothetical cartoon scheme of NM-mechanotransduction events caused by nuclear deformation. Known types of NM mechanotransduction mechanisms are highlighted by gray shaded circles. INM-tension promotes the adsorption of peripheral membrane proteins with protruding hydrophobic groups (blue, cPLA_2_; yellow, farnesylated Lamin B). Membrane tension is spatially propagated from the INM to the ONM/ER by lipid flow and associated with weak (upon swelling, left side of cartoon) or strong (upon compression, right side of cartoon) Ca^2+^ influx from the ER lumen through opening of mechanosensitive ion channels. Cell compression but not swelling promotes yes-associated protein (YAP) accumulation within the nucleus, perhaps through mechanical regulation of nuclear pore permeability. For further explanations, please see the main text.

Although current structural imaging techniques make this difficult to observe, the sequence of events may be envisioned as follows (Fig. 2): Upon stretch, the phospholipid headgroups in the bilayer move apart, and the equilibrium lateral lipid pressure of the bilayer decreases. This gives rise to voids between lipid headgroups (“lipid packing defects”) into which protein residues can insert. Depending on their individual insertion depth, peripheral membrane proteins will reach optimal membrane interactions at different equilibrium lateral lipid pressures.^28^ More tension is required to accommodate the insertion of deeply penetrating domains compared to shallowly interacting ones. Compared to related domains, the C2-domain of cPLA_2_ inserts more deeply into the bilayer, which likely explains its exceptional sensitivity to membrane tension.^29–32^ Interestingly, sensing of membrane curvature or conical lipid content by proteins with amphipathic helices or lipid anchors follows an analogous, if not identical principle.^33–38^ Here, the lipid packing defects that promote peripheral protein adsorption are generated by the angles between neighboring lipids, or conical lipid shapes—not stretch.

The “force through lipid” paradigm of ion channel mechanotransduction^39^ long-reigned as the sole-studied mechanism of membrane [mostly plasma membrane (PM-)] tension sensing. OS first highlighted the importance of peripheral membrane proteins as physiological membrane tension sensors, fueling the idea of the NM as additional mechanosensory surface of the cell.

## BEYOND OS

Cell and organelle swelling are hallmark features of necrotic cell death. Pathologists refer to them as cytotoxic or cellular edema (oidein, Greek for “to swell”)^40^ as opposed to interstitial edema, where fluid accumulates between cells. In a recent study of iron- and lipid peroxide-dependent necrosis (“ferroptosis”), fluorescent zebrafish cPla_2_ was employed as a reporter for pre-lytic nuclear swelling and NM-tension. cPla_2_ translocation to the inner NM was observed well before cells underwent osmotic lysis.^41^ Given cPLA_2_'s essential role in the synthesis of inflammatory lipids, it is conceivable that necrotic cells with the appropriate enzymatic repertoire can alert the immune system by secreting inflammatory eicosanoids while undergoing osmotic swelling. Such pre-lytic, biomechanical danger signals may ring the alarm bell well before conventional damage associated molecular patterns are released by cell lysis.

In addition to cPLA_2_, there may be other peripheral membrane proteins regulated by NM-tension. For example, 5-lipoxygenase has also been shown to be tension-sensitive.^25,27^ This enzyme contains a Ca^2+^ dependent PLAT (“polycystin-1, lipoxygenase, alpha-toxin”) membrane-binding domain that is functionally analogous to the C2-domain of cPLA_2_. Vertebrate genomes contain more than ∼150 “C2-like” domains, some of which mediate the membrane interactions of important signaling proteins such as protein kinase C and others. Additionally, proteins with lipid-anchors (e.g., farnesylation, palmitoylation, etc.) or amphipathic helices (e.g., ALPS-motifs) may be also sensitive to bilayer tension given their known tendency to insert into lipid packing defects caused by membrane curvature (e.g., at nuclear pores or poles) and conical lipids.^27,36,37^ Several nuclear proteins, including lamins and nuclear pore complex components, contain such motifs. For example, the farnesylation of B-type lamins may render them sensitive to curvature, stretch, and lipid composition. Indeed, the concentration of lamin B1 was shown to be decreased at nuclear blebs and at the negatively curved poles of flat nuclei.^42^ In these regions, lipid headgroup packing is expected to be denser and, thus, more prohibitive for hydrophobic insertion than in other parts of the NM. Although the observations by Nmezi and colleagues raise the intriguing possibility that farnesylated lamin B1 is curvature- and maybe stretch-sensitive akin to cPLA_2_, there are plausible, alternative explanations: For one, owing to steric constraints, lamin B1 networks may not assemble as efficiently at highly negatively curved surfaces. Future work should distinguish between these possibilities. Furthermore, the potential mechano-sensing and -transduction roles of other peripheral NM proteins with pronounced hydrophobic or amphipathic features should be systematically evaluated.

## AN INNER SENSE OF SPACE AND PRESSURE

Like swelling, compression is another type of nuclear deformation that cells frequently experience *in vivo* [e.g., when traversing narrow tissue channels or squeezing through pores in an extracellular matrix (ECM) or vessels]. Given its size and stiffness, the nucleus limits cell migration through such confined spaces^43^ as strong nuclear confinement can result in nuclear- and DNA-damage.^44,45^ If fast migrating cells, such as leukocytes, have the choice between a route that involves nuclear confinement and one that does not, they will choose the path of least resistance.^46^ Interestingly, various cell types switch from mesenchymal-like 2D migration to amoeboid-like 3D motility upon confinement.^47–52^ Two recent studies suggest that this switch is triggered when critical deformation stretches the NM.^53,54^ Floppy nuclei with large membrane reservoirs or a fragile lamina require stronger confinement to develop NM-tension. NM-tension, in turn, leads to cPLA_2_ activation and release of AA. Through a mechanism that is not well understood, AA initiates cortical actomyosin contractions and PM-blebbing, which can drive cell movements.^53,54^ Thus, NM-mechanotransduction controls confined cell migration with potential implications for immune cell migration and cancer metastasis.

## CHANNELING NUCLEAR MEMBRANE TENSION

Ca^2+^ is a second messenger that regulates a plethora of cellular pathways, including wound detection and the above-mentioned eicosanoid cascade, downstream of chemical or mechanical stimulation. Although mechanosensitive Ca^2+^ signaling is typically thought to be mediated by ion channels located on the plasma membrane, recent work suggests that at least one of these channels, Piezo1, can also cause Ca^2+^ efflux from the ER upon nuclear deformation by repeated cell stretching.^55^ Through this mechanism, epidermal progenitor cells can adjust their physical chromatin resistance to repeated mechanical challenge thereby avoiding DNA damage.^55^ The Ca^2+^ fluxes were shown to depend on lamin A levels, that is, they seemed to be coupled to nuclear mechanics. It will be interesting to see whether this mechanism applies to other frequently stretched cell types with intracellular Piezo1 channels such as vascular smooth muscle cells.^56^ Other studies that observed mechanosensitive Ca^2+^ signals from intracellular stores upon nuclear deformation^53,54^ ruled out a central role for Piezo1 based on pharmacologic evidence. Instead, they hypothesized that mechanosensitive InsP_3_R- or Stromal Interaction Molecule (STIM)-Orai-dependent mechanisms are involved.

Different types of nuclear deformations differ in their ability to elicit Ca^2+^ transients: Compression leads to stronger Ca^2+^ signals than swelling.^54^ If swelling and compression stretch the NM with similar magnitude and timing, other cues besides, or instead of, bilayer tension must control intracellular Ca^2+^ release when nuclei are deformed. According to the “force from filament” paradigm,^39^ mechanosensitive channels are also regulated by cytoskeletal linkages and tension. Consistent with this idea, cell-extracellular matrix (ECM) attachments modulate the sensitivity of Piezo1 channels to push and pulling forces.^57^ The integration of bilayer tension, ECM-attachment, and cytoskeletal forces may allow cells to sense nuclear deformation vectors in a nuanced way, enabling them to distinguish between different nuclear shapes or “postures.”^54^ In addition to NM-tension, topological rearrangements of intracellular membranes, such as the alteration of ER-plasma membrane proximity,^54^ may contribute to the differences in intracellular Ca^2+^ fluxes observed between cell swelling and compression. Finally, differences in cell-adhesion and cell-volume signaling may help to establish the shape-dependence of mechanosensitive Ca^2+^ fluxes.

Interestingly, nuclear shape sensitivity is also observed in other instances of nuclear mechanotransduction. The nuclear pore complex is receiving increased attention as a dilatable and contractable gate keeper of the nucleus.^58–60^ Compression, but not swelling, causes the nucleoplasmic accumulation of the mechanosensitive transcription factor yes-associated protein (YAP) (Fig. 2), at least in part, through mechanical regulation of nuclear pore permeability.^61^ Apparently, YAP can distinguish between swollen and squeezed nuclei, but how? Whether this points to a common principle of nuclear pore and ion channel regulation upon nuclear deformation remains to be determined. A very recent study used cryo-electron tomography to show that nuclear pores of yeast cells contract upon hypertonic shock and energy depletion, indicating that their diameter is regulated by NM-tension.^60^ If the same mechanical behavior applies for mammalian nuclear pores, it will be interesting to next ask whether or how mechanosensitive changes in nuclear pore diameter contribute to the nuclear accumulation of YAP and other transcription factors.

## THE ENIGMA OF NUCLEAR MEMBRANE MECHANICS

It is little studied and understood how the NM, with its complex cytoskeletal, lamina, and chromatin linkages, behaves upon nuclear deformation. Comparably sized artificial membrane models, such as GUVs, despite their far simpler membrane topology and composition, can still provide some basic insights. When a GUV is swollen or squeezed, membrane tension rises once all undulations and invaginations are fully unfolded.^27^ A decrease in lipid packing allows the membrane to expand by an additional ∼5%–10% when stretched by micropipette aspiration or osmotic pressure, respectively.^62,63^ The lytic area strain, in part, depends on the lipid composition. For example, GUV membranes can be strengthened by inclusion of cholesterol,^62^ although this stabilization effect also depends on the other lipid components of the membrane.^64,65^ Cholesterol is thought to act like a “glue” between the phospholipids increasing their packing order. The PM contains more cholesterol, higher levels of saturated lipids, and more negatively charged phospholipids (in the inner leaflet) than ER membranes.^66^ As the ER is continuous with the NM, and lipids diffuse quickly, the lipid composition of the ER likely approximates NM lipid composition. If this assumption is correct, the NM is expected to be less stable than the PM, while being more prone to developing lipid packing defects that can serve as hydrophobic adsorption sites for peripheral membrane proteins.^67^ Attributing membrane behavior to lipid composition alone is, of course, an oversimplification. Integral membrane proteins, their coupling to underlying cytoskeletal structures, as well peripheral membrane protein adsorption likely also play an important role. So far, the question of how this added complexity affects membrane mechanics has been only studied for the PM.^68^ This needs to be extended to the NM, which, unfortunately, is less accessible to direct physical probing techniques such as micropipette aspiration, atomic force microscopy, or optical traps.

In addition to facilitating peripheral membrane protein adsorption (e.g., of cPLA_2_), lower lipid packing promotes membrane fusion. By tension-induced insertion of smaller membrane particles, artificial and native membranes can stabilize this tension below lytic levels to avoid rupture.^69–71^

As with GUVs, membrane unfolding/smoothing followed by tension is also observed when intact nuclei deform.^53^ Osmotic swelling increases the NM surface area by ∼60%,^72^ which is more than obtainable by lowering lipid packing alone. Unfolding of nucleoplasmic reticulum (NR) invaginations prevent NM stretch in response to small, “non-critical” deformations. As a result, floppier nuclei with a lot of membrane invaginations have a higher threshold for activating NM-mechanotransduction.^53^ Given their size, number, and ability to dilate and contract,^59,60^ nuclear pores may also contribute to nuclear surface regulation during mechanical stress. As the ER is an evagination of the outer NM, can it just “unfold” into the nuclear envelope? Assuming that the ER-NM continuity is unaffected by mechanical perturbation and that the overall membrane system behaves like a heavily invaginated GUV, NM-tension could arguably only rise after the ER has fully collapsed into the NM, which has yet to be observed. Alternatively, if the ER resists collapse, additional nuclear surface may be gained by membrane flow down a lateral lipid pressure gradient from a relatively relaxed ER into a tense NM (Fig. 2). Such tension-mediated membrane flows have been studied in other parts of the cell^73,74^ and are involved in surface area regulation of cells and organelles.^75^ ER-to-NM membrane flow may help the nucleus prevent membrane rupture by maintaining a sub-lytic steady-state membrane tension during persistent, critical nuclear deformation. Likely, this membrane flow will be not only determined by the respective lateral lipid pressure (or tension) gradient but also by the architecture of the NM and its density of immobilized, integral proteins.^76–78^ Akin to pillars in a stream, integral NM-proteins coupled to the extranuclear cytoskeleton, the nuclear lamina, or chromatin can slow down lipid flow to limit the spatial propagation of membrane tension within the NM–ER membrane continuum. Consistent with this idea, deletion of the NM-protein Lem2 and the ER-membrane protein Lnp1 was shown to increase membrane flow into and out of the yeast nucleus in response to altered membrane synthesis and nucleocytoplasmic transport.^79^ Other abundant NM-proteins, once immobilized on some intra- or perinuclear structure, may have similar flow barrier-like functions. These speculations enter the largely uncharted territory of NM mechanics.

## THE ROAD LESS TRAVELLED

The PM has been intensely studied as a main mechanosensitive surface of the cell. Over the last six years, a new notion of the NM as additional mechanosensory surface has emerged. A lack of methods to measure membrane mechanics of intracellular organelles has made investigating NM-mechanotransduction a challenge to date. Yet, there are promising membrane tension dyes being developed,^80^ and the discovery of peripheral membrane proteins as physiological tension sensors will likely inspire the design of genetically encoded reporters that allow imaging NM-tension in live cells and intact animals. To fully comprehend how cells control their NM-tension, the mechanics of all attached nuclear components and their mechanical interconnections must be examined. This could involve measuring the forces exerted on nuclear envelope proteins (e.g., nesprins) and chromatin, for example, with fluorescence resonance energy transfer (FRET) reporters.^81^ The next bricks that will pave the way to a better understanding of NM-mechanotransduction are in sight. Let us paint them yellow and travel the road!

## Data Availability

Data sharing is not applicable to this article as no new data were created or analyzed in this study.
